# Supplementary material for: A novel approach for longitudinal analysis of serum biomarkers of joint metabolism and knee injury in military officers
Source: PLoS One. 2026 Jan 30;21(1):e0341836. doi: 10.1371/journal.pone.0341836 (PMC12857958; doi:10.1371/journal.pone.0341836)

**Supplementary Figure S1. Distribution of biomarker levels at matriculation and graduation after shifted log transformation.** The red and blue curves indicate the kernel density estimate for the distribution of biomarker levels colored by sex (male=red, female=blue). **CTX:** C-terminal telopeptides of type I collagen; **NTX:** N-terminal telopeptides of type I collagen; **CPII:** C-terminal propeptide of Type II collagen; **C12C:** Collagen Type I and II collagenase-generated cleavage epitopes; **C2C:** Collagen Type II collagenase-generated cleavage epitopes**; Mat:** Matriculation; **Grad:** graduation.


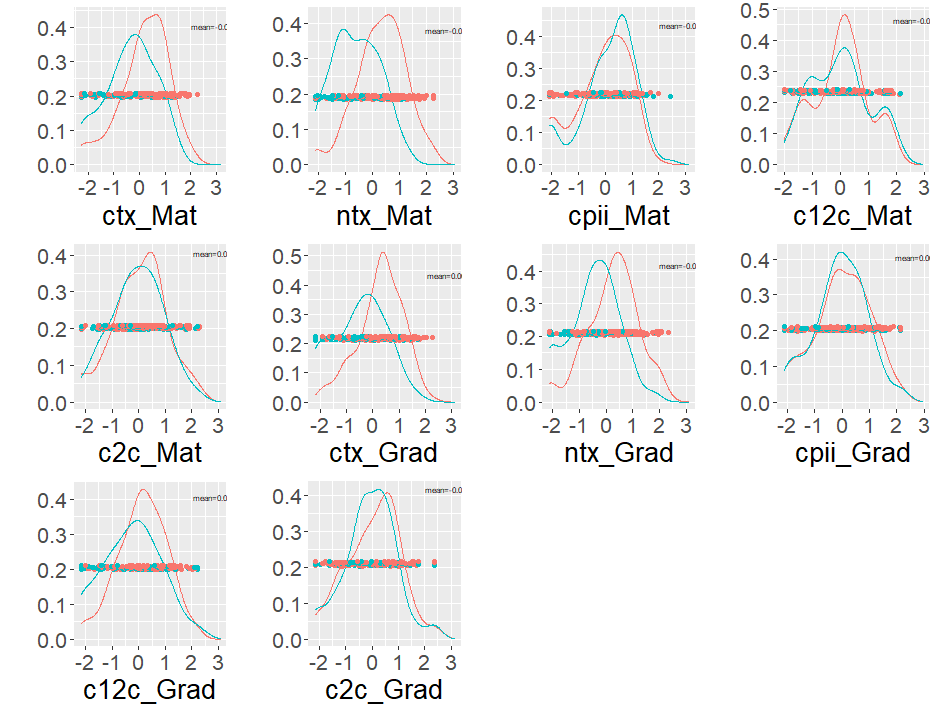

Supplement: S1 Fig — The red and blue curves indicate the kernel density estimate for the distribution of biomarker levels colored by sex (male=red, female=blue). CTX: C-terminal telopeptides of type I collagen; NTX: N-terminal telopeptides of type I collagen; CPII: C-terminal propeptide of Type II collagen; C12C: Collagen Type I and II collagenase-generated cleavage epitopes; C2C: Collagen Type II collagenase-generated cleavage epitopes; Mat: Matriculation; Grad: graduation. (DOCX) [file pone.0341836.s001.docx]
