# Supplementary material for: A novel approach for longitudinal analysis of serum biomarkers of joint metabolism and knee injury in military officers
Source: PLoS One. 2026 Jan 30;21(1):e0341836. doi: 10.1371/journal.pone.0341836 (PMC12857958; doi:10.1371/journal.pone.0341836)

**Supplementary Figure S2. First shared AJIVE direction of variation in MEN without knee ligament/meniscal injury at matriculation.** X axes show the amount that each biomarker and clinical variable contributed to the first mode of variation. Biomarker levels at matriculation and graduation were transformed without adjustment on age. **CTX:** C-terminal telopeptides of type I collagen; **NTX:** N-terminal telopeptides of type I collagen; **CPII:** C-terminal propeptide of Type II collagen; **C1,2C:** Collagen Type I and II collagenase-generated cleavage epitopes; **C2C:** Collagen Type II collagenase-generated cleavage epitopes**; BMI:** Body Mass Index; **Mat:** Matriculation; **Grad:** graduation.


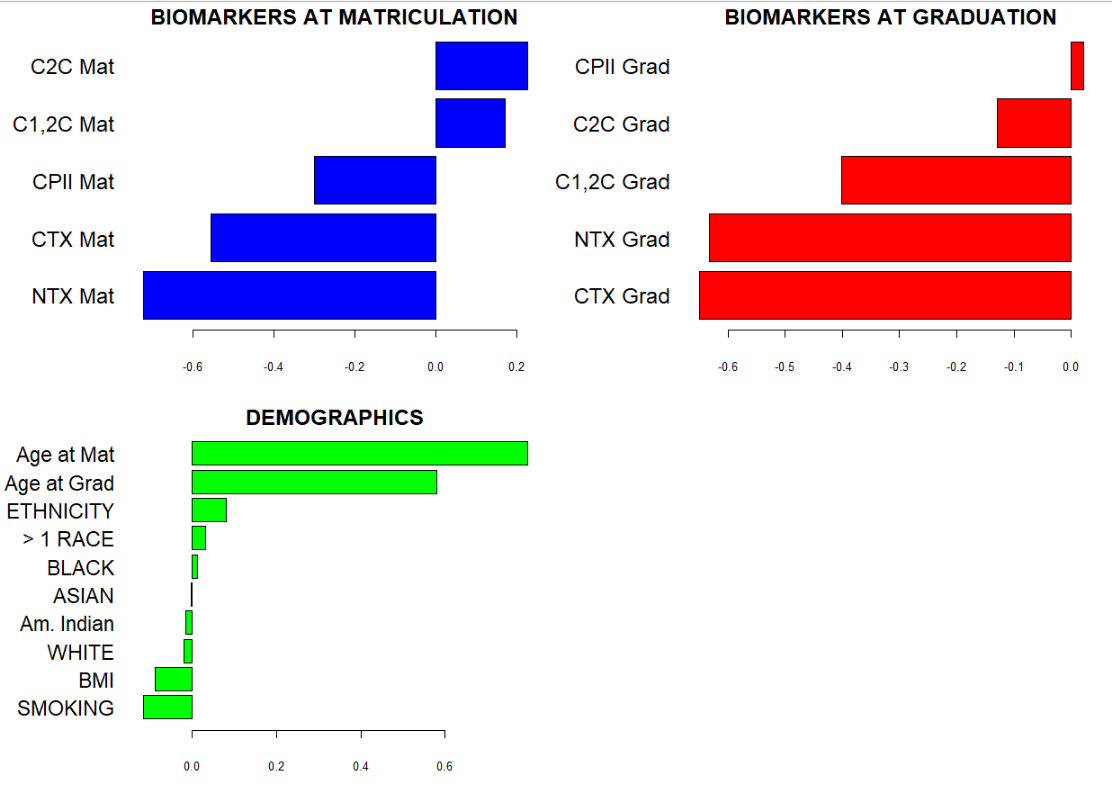

Supplement: S2 Fig — X axes show the amount that each biomarker and clinical variable contributed to the first mode of variation. Biomarker levels at matriculation and graduation were transformed without adjustment on age. CTX: C-terminal telopeptides of type I collagen; NTX: N-terminal telopeptides of type I collagen; CPII: C-terminal propeptide of Type II collagen; C1,2C: Collagen Type I and II collagenase-generated cleavage epitopes; C2C: Collagen Type II collagenase-generated cleavage epitopes; BMI: Body Mass Index; Mat: Matriculation; Grad: graduation. (DOCX) [file pone.0341836.s002.docx]
