# Supplementary material for: A novel approach for longitudinal analysis of serum biomarkers of joint metabolism and knee injury in military officers
Source: PLoS One. 2026 Jan 30;21(1):e0341836. doi: 10.1371/journal.pone.0341836 (PMC12857958; doi:10.1371/journal.pone.0341836)

**Supplementary Figure S3. First 3 directions representing individual variation in biomarker levels at matriculation (ICm) in WOMEN that are not related to the biomarker levels at graduation and participant features.**


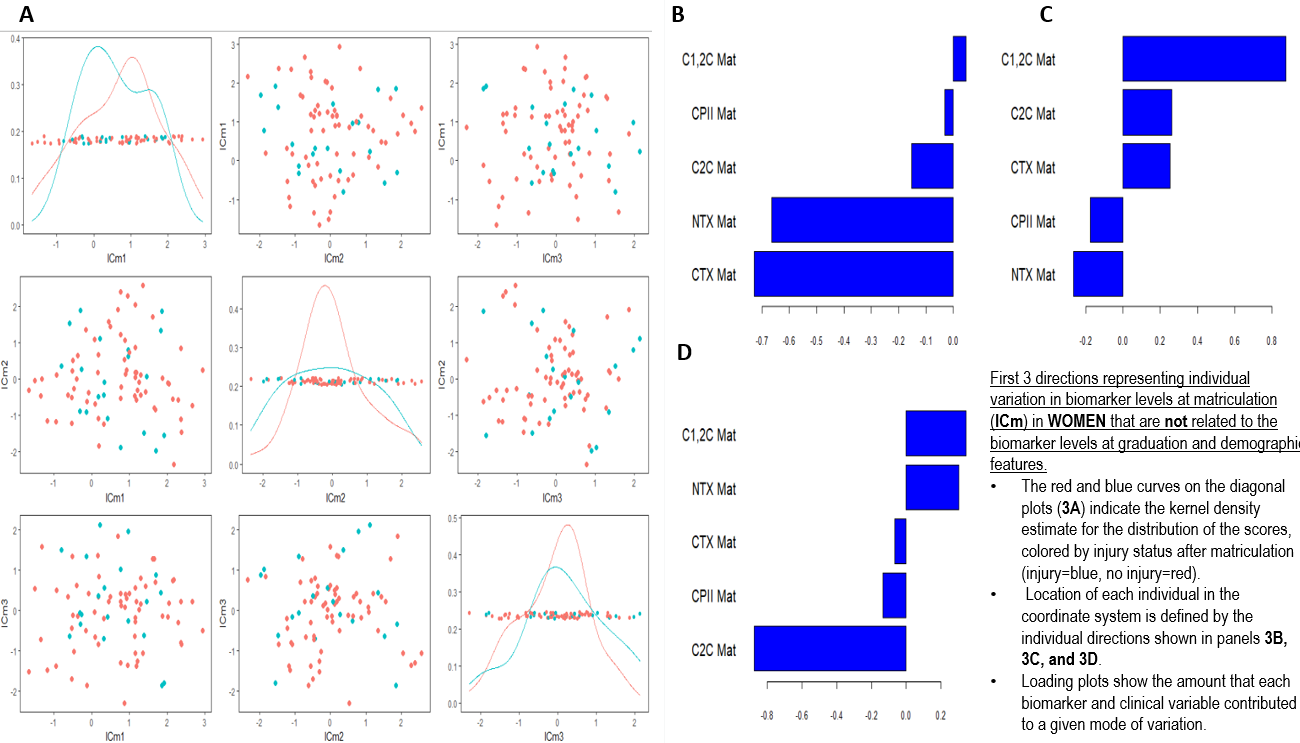

Supplement: S3 Fig — (DOCX) [file pone.0341836.s003.docx]
