# Supplementary material for: A novel approach for longitudinal analysis of serum biomarkers of joint metabolism and knee injury in military officers
Source: PLoS One. 2026 Jan 30;21(1):e0341836. doi: 10.1371/journal.pone.0341836 (PMC12857958; doi:10.1371/journal.pone.0341836)
Supplement: S1 File — (PPTX) [file pone.0341836.s005.pptx]

## Slide 1
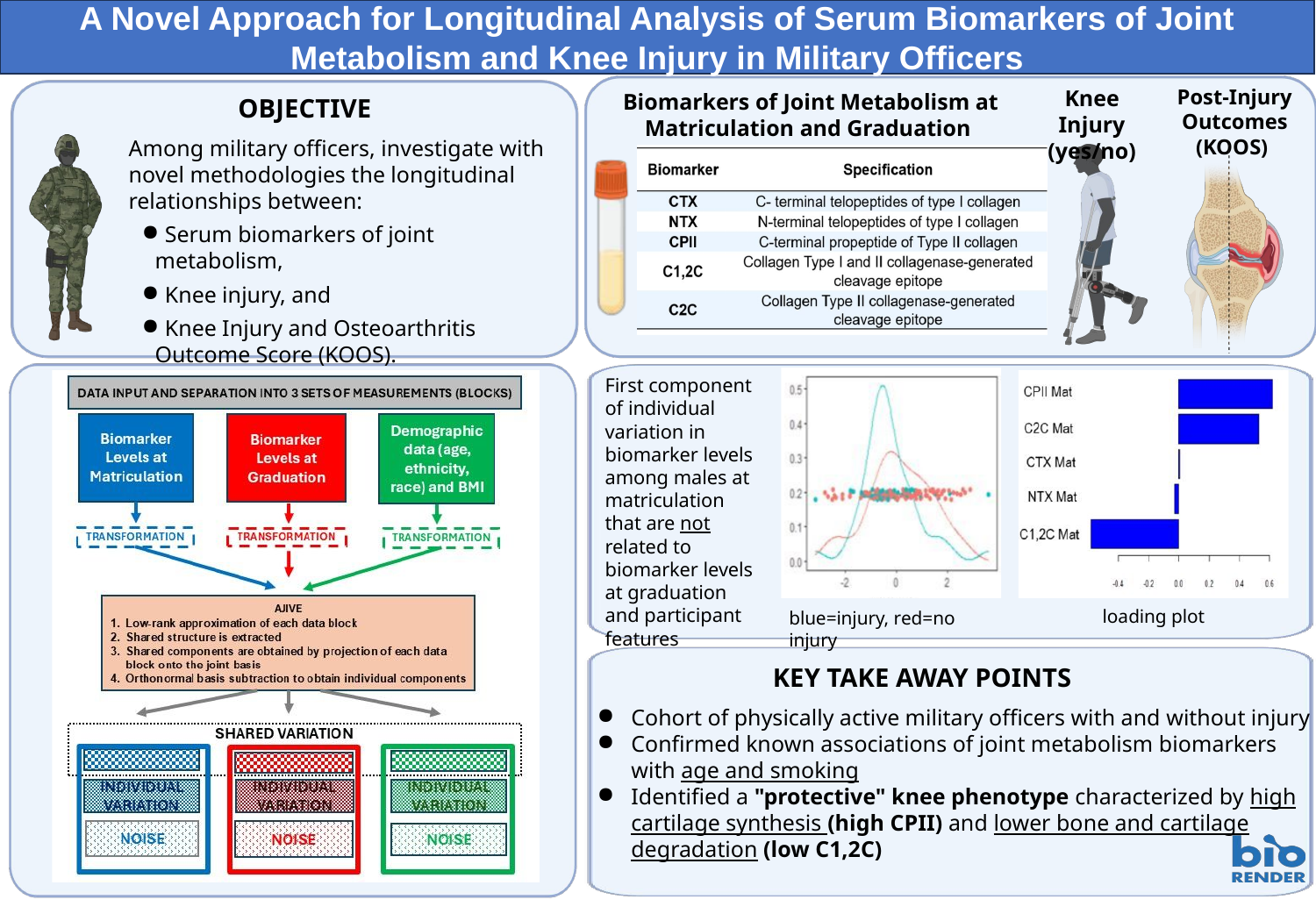

A Novel Approach for Longitudinal Analysis of Serum Biomarkers of Joint Metabolism and Knee Injury in Military Officers
Post-Injury Outcomes (KOOS)
Knee Injury (yes/no)
Biomarkers of Joint Metabolism at Matriculation and Graduation
OBJECTIVE
Among military officers, investigate with novel methodologies the longitudinal relationships between:
 Serum biomarkers of joint metabolism,
 Knee injury, and
 Knee Injury and Osteoarthritis Outcome Score (KOOS).
First component of individual variation in biomarker levels among males at matriculation that are not related to biomarker levels at graduation and participant features
loading plot
blue=injury, red=no injury
KEY TAKE AWAY POINTS
Cohort of physically active military officers with and without injury
Confirmed known associations of joint metabolism biomarkers with age and smoking
Identified a "protective" knee phenotype characterized by high cartilage synthesis (high CPII) and lower bone and cartilage degradation (low C1,2C)
